# Recombinant Antibodies against Mycolactone

**DOI:** 10.3390/toxins11060346

**Published:** 2019-06-17

**Authors:** Leslie Naranjo, Fortunato Ferrara, Nicolas Blanchard, Caroline Demangel, Sara D’Angelo, M. Frank Erasmus, Andre A. Teixeira, Andrew R.M. Bradbury

**Affiliations:** 1 Specifica Inc., Santa Fe, NM 87505, USA; lnaranjo@specifica.bio (L.N.); fferrara@specifica.bio (F.F.); sdangelo@specifica.bio (S.D.); ferasmus@specifica.bio (M.F.E.); ateixeira@specifica.bio (A.A.T.); 2 Université de Haute-Alsace, Université de Strasbourg, CNRS, LIMA, UMR 7042, 68000 Mulhouse, France; n.blanchard@unistra.fr; 3 Immunobiology of Infection Unit, Institut Pasteur, INSERM U1221, 75011 Paris, France; caroline.demangel@pasteur.fr; 4 New Mexico Consortium, Los Alamos, NM 87544, USA

**Keywords:** mycolactone, Buruli ulcer, recombinant antibody, phage display, yeast display, single chain Fv

## Abstract

In the past, it has proved challenging to generate antibodies against mycolactone, the primary lipidic toxin A of *Mycobacterium ulcerans* causing Buruli ulcer, due to its immunosuppressive properties. Here we show that in vitro display, comprising both phage and yeast display, can be used to select antibodies recognizing mycolactone from a large human naïve phage antibody library. Ten different antibodies were isolated, and hundreds more identified by next generation sequencing. These results indicate the value of in vitro display methods to generate antibodies against difficult antigenic targets such as toxins, which cannot be used for immunization unless inactivated by structural modification. The possibility to easily generate anti-mycolactone antibodies is an exciting prospect for the development of rapid and simple diagnostic/detection methods.

## 1. Introduction

Buruli ulcer (BU) is a disfiguring and debilitating neglected tropical disease caused by *Mycobacterium ulcerans*, a slow-growing organism that produces mycolactone, a unique lipidic toxin essential to the pathogenesis of BU [1,2,3,4,5,6], and unique to *M. ulcerans*. In addition to causing skin necrosis, mycolactone causes cytotoxicity and immunosuppression [5,7,8,9,10,11,12], which may prevent *M. ulcerans* clearance by the host. Mycolactone exerts its toxicity by inhibiting the translocation of secretory and membrane proteins across the ER membrane, by inhibiting the heterotrimeric Sec61 complex [13]. Interestingly, the ex vivo cytotoxic potency of mycolactone in biopsies from BU lesions appears to be greater than that of corresponding amounts of purified mycolactone, suggesting that in vivo the toxin exists in biological complexes that exacerbate its toxicity. BU continues to develop in West Africa [14,15], Australia [16,17], and other areas [18,19]. Transmission is presumed to involve cutaneous inoculation through skin injuries and/or insect bites [17,20]. The disease begins as a painless nodule or plaque, which subsequently ulcerates and grows inexorably, eventually threatening limbs and causing functional disabilities, often resulting in permanent social, economic, and developmental problems. The mortality rate of BU is low and usually related to untreated cases [21]. Ulcers are characteristically painless and may lead to bacterial super-infection and sepsis. Even appropriately treated large ulcers spanning joints often heal with disabling scarring and limb contracture [22].

Until 2004, treatment of BU consisted of wide surgical excision and skin grafting. At that time the WHO [23] first recommended therapy with rifampin and streptomycin, based on pioneering studies in the mouse footpad model [24,25,26] and confirmed by subsequent clinical experience [27,28]. Early implementation of this pharmaceutical approach eliminates the need for surgery and its disabling sequelae, making early diagnosis crucial. However, no rapid, simple, specific, non-invasive point-of-care (POC) diagnostic test exists. Available tests, including microscopy, culture, PCR [29], fluorescent thin layer chromatography [30] and the histopathology of lesions all require relatively sophisticated laboratory facilities and expertise [31]. The problem is aggravated by the complex differential diagnosis, which depends upon the clinical presentation (ulcer or nodule), and geographical localization.

Most POC diagnostics, particularly lateral flow assays [32], rely on antibodies as detection agents. However, given the cytotoxicity and immunosuppressive effects of mycolactone [5,7,8,9,10,11,12], the generation of antibodies by traditional immunization has, until recently [33], been extremely challenging. This problem was overcome by immunizing with a truncated synthetic non-toxic variant [33], leading to the derivation of a number of murine monoclonal antibodies showing in vitro neutralization activity. In humans, subjects with active disease show strong humoral reactions to some *M. ulcerans* proteins [34], including culture filtrate proteins [35], but with high cross-reactivity to antigens from other mycobacterial species [34], with up to 37% of control subjects showing positive antibody responses [35,36]. No systematic study investigating the antibody response to the toxin appears to have been published.

An alternative to the use of traditional immunization to generate antibodies has been the use of in vitro display methods [37] to select antibodies from large naïve or immune libraries. In these techniques, large antibody libraries in either the Fab or single chain Fv (scFv) format [38] are displayed on the surface of filamentous phage [39,40] or yeast [41,42], and antibodies binding targets of interest are purified away from the multitude of non-binding antibodies. Targets are usually immobilized and the binding antibodies separated from non-binding antibodies by a series of washing steps, followed by elution. The selection of antibodies from vast antibody libraries, instead of using animal immunization and hybridoma technology, offers the advantage of guiding the selection process towards antibodies with rare specificities, epitopes or characteristics that are extremely difficult to find with animal-based methods. The key feature of in vitro display systems is that phenotype (the displayed antibody) and genotype (the gene encoding the displayed antibody) are coupled in such a way that selection of an antibody on the basis of its binding activity leads to simultaneous selection of the gene that encodes it. This guarantees the permanent availability of selected clones over time, by virtue of gene synthesis, and also makes it possible to express clones in different antibody formats (e.g., with Fc domains from different species), as well as improve antibody properties, such as affinity. Furthermore, recombinantly expressed antibodies have been shown to be more specific than their hybridoma expressed counterparts, which often express additional immunoglobulin chains that detract from their specificity [43].

In general, the larger the library, the higher the affinity of antibodies that can be selected, although this tends to be target specific, with protein targets generally yielding much higher affinities than small targets such as mycolactone. We developed a recombinatorial method [44,45] to create particularly large phage antibody libraries, from which human antibodies against a number of challenging targets have been generated [46,47,48]. Here we describe the application of this approach to select human antibodies recognizing mycolactone.

The aim of the present study was to generate a set of human antibodies, specifically recognizing mycolactone, that could potentially provide a set of novel molecular tools for the study, diagnosis, prophylaxis, and potential treatment of BU.

## 2. Results

### 2.1. Antibody Selection

**Figure 1:**
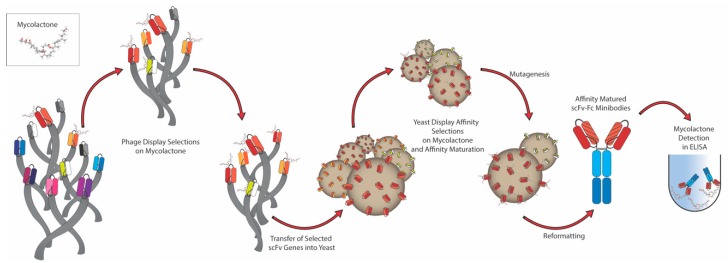
Strategy used to select antibodies using both phage and yeast display. Antibodies are first selected against mycolactone using two rounds of phage display, after which the whole selection output is cloned into a yeast display vector. A further one or two rounds of sorting by flow cytometry allow the subsequent isolation and testing of single clones, followed by affinity maturation by mutagenesis to select for higher affinity binders. The final selected antibodies are finally expressed as ‘scFv-Fc fusions’—where the variable domain of the antibody (scFv) is fused with the CH2-CH3 constant region (Fc) of human immunoglobulin IgG1—and further validated in an enzymatic assay (ELISA) for its specific binding to the toxin of interest.

The general strategy used to generate recombinant antibodies against a challenging target such as mycolactone has been summarized in the schematic diagram shown in Figure 1.

**Figure 2:**
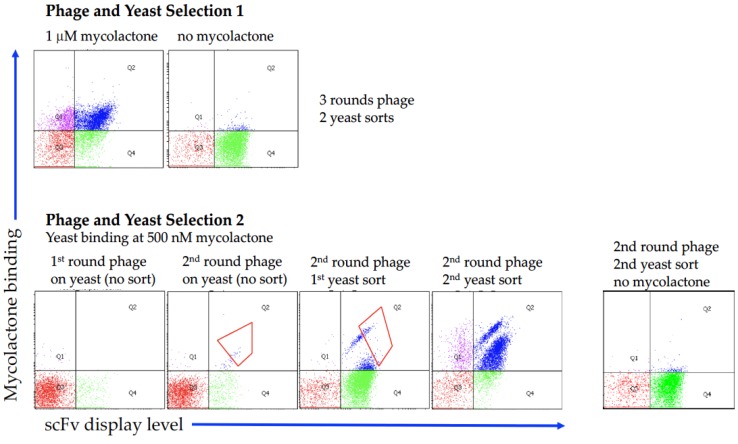
Analysis of the flow cytometric output of scFv selections displayed on the surface of yeast. Selection 1 was carried out using plastic containers, while Selection 2 was carried out using glass containers. For Selection 2 the enrichment progress during the sorting steps is shown, including the gates used for the sorting of binding cells. The populations labeled as “no mycolactone” show the background binding for the fluorescently conjugated streptavidin.

Biotinylated mycolactone was used to select antibodies from a large well-characterized phage single chain Fv (scFv) antibody library [44,49] using a combination of phage and yeast display, which combines the advantages of phage display (selection from extremely large libraries) with those of yeast display (precise selection calibration by flow cytometry) [46,50]. Figure 2 shows the representative flow cytometry plots of selection outputs, in which after phage were selected using an excess of biotinylated mycolactone, the enriched antibodies were subcloned into the yeast display format and sorted at 1 μM for 1–2 rounds. Each dot represents an individual yeast organism displaying 30,000–100,000 copies of a single antibody. The *x*-axis shows the level of antibody displayed on the surface of the yeast, as recognized by a fluorescently labeled antibody specific to the SV5 tag [51,52], fused in frame with the displayed single chain Fv antibodies. The *y*-axis shows the amount of bound biotinylated mycolactone, detected using fluorescently labeled streptavidin, with greater binding indicated by increased values on the *y*-axis. Yeast displaying desirable antibodies are to be found in the upper right quadrant, representing those that display antibodies and bind to mycolactone. Those found in the lower left quadrant represent daughter cells known to lose antibody display immediately after cell division.

**Figure 3:**
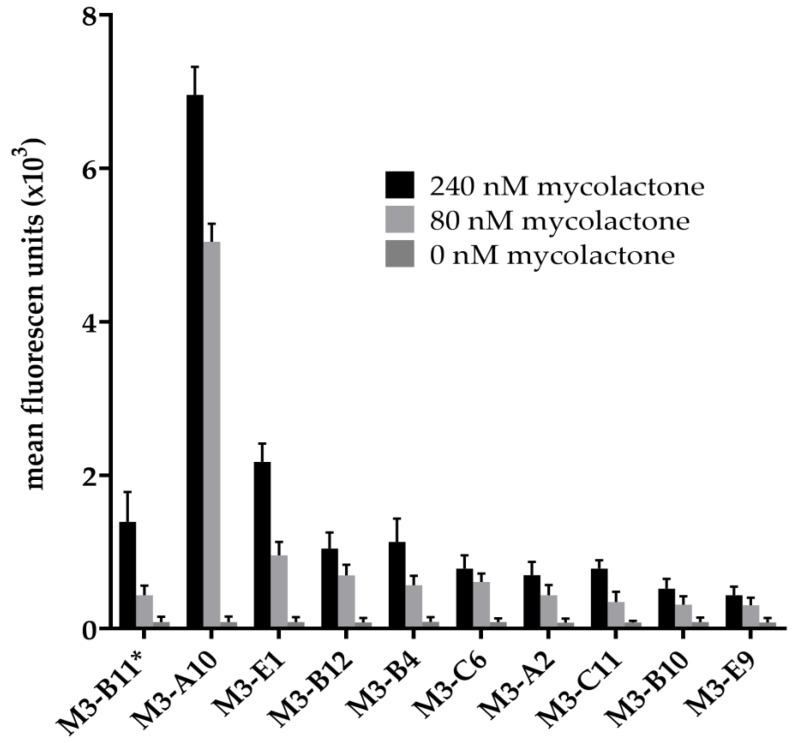
Binding profile analysis by Flow cytometry of individual selected scFvs tested against 240 and 80 nM mycolactone and a negative control. M3_B11 was the only clone identified in Selection 1 and among the 10 identified after Selection 2.

In a first selection attempt (Selection 1, Figure 2), the phage antibody library was incubated with 5 µM biotinylated mycolactone and selection was carried out using streptavidin coated magnetic beads and an automated system (Kingfisher System, Thermo Fisher Scientific, Waltham, MA, USA). When 96 individual yeast clones were isolated, and their V gene sequenced, clone M3_B11 (Table 1) was the only clone found. We concluded that this result may have been due to the known hydrophobicity of mycolactone, and the fact that the Kingfisher system uses plastic containers to carry out selections, with probably most of the mycolactone adsorbed to the plastic surface and not available for selection. Consequently, a second selection (Selection 2, Figure 2) was carried out manually in glass vials (Agilent Technologies, Santa Clara, CA, USA) using 500 nM of biotinylated mycolactone in each phage selection round, and subsequently following the same approach by subcloning the phage selection output into the yeast display vector and carrying out the sorting steps using 1 μM antigen. During the sorting process two populations were noticeable after the first enrichment step with noticeably different affinities for the binders. When setting the gate for the sorting we included both of these populations. In this case, when 96 single yeast clones were isolated and sequenced we were able to recover a total of 10 different antibodies, including the only one selected in the previous selection campaign. Table 1 illustrates the 10 different antibodies, with two antibodies (M3_A10 and M3_B11) differing by only a single amino acid, and two others (M3_B10 and M3_B12) differing only in the HCDR3s. All the different clones were tested for binding to mycolactone by flow cytometry at two concentrations (240 nM and 80 nM), and shown to bind mycolactone (Figure 3), albeit with poor affinities, with the majority of the clones estimated to have Kds >1 μM.

### 2.2. Affinity Maturation

**Figure 4:**
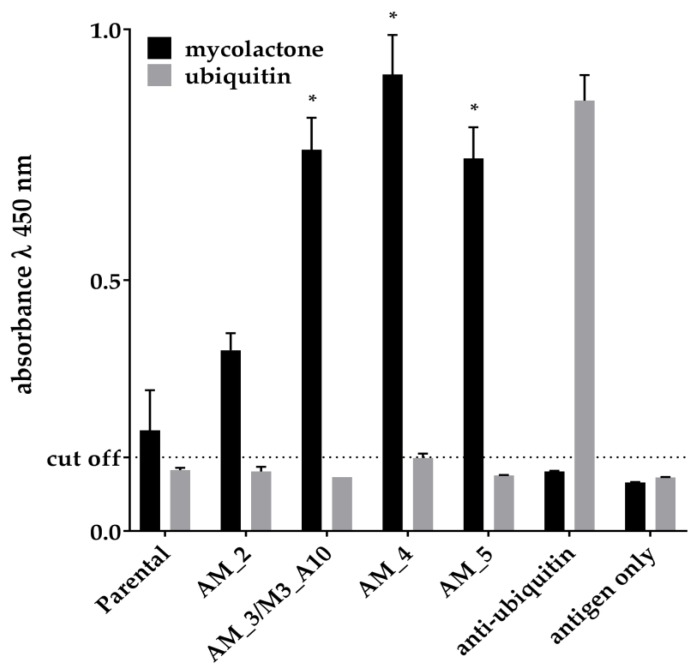
Detection activity of the scFv-Fc derived from individual affinity matured scFvs tested by ELISA against 300 nM biotinylated mycolactone. Ubiquitin (300 nM) was used as negative control antigen and an anti-ubiquitin scFv-Fc derived from the same library was used as an assay positive control. * Indicate a *p* value < 0.05 compared to the parental clone.

The clone M3_B11, which was found after the first selection effort—Selection 1—was initially taken forwards for further analysis and affinity maturation. We tried to improve the binding activity by introducing random mutations by error prone PCR (ePCR) [53]. A population of mutated clones was obtained and displayed on yeast, and subsequently enriched for improved affinity using four steps of fluorescence activated cell sorting. Eventually, 96 affinity matured clones were picked, sequenced and tested, and 6 different mutated clones were isolated by Sanger sequencing. Table 2 shows the sequences of improved clones and their affinities as measured on the yeast surface. Among the matured variants, one was found to be identical to M3_A10, selected independently using the Selection 2 approach. By carrying out antigen titrations on these antibodies displayed directly on the yeast surface and assessing the mean fluorescence intensity of the yeast populations it was possible to calculate antibody affinities [54], which ranged from 145 nM to 470 nM. These clones were recloned into a yeast secretion vector for expression as scFv fused to the Fc domain of IgG1 immunoglobulin and tested for binding to mycolactone in an ELISA format, thanks to the presence of the Fc portion (CH2-CH3 domains of a human IgG1) that is detectable with commonly available secondary anti-human IgG reagents [50]. The ELISA results in Figure 4 are directly comparable to the affinities listed in Table 2: the parental clone, the one with the worse affinity, was characterized by a lower detection activity, while the improved clones performed better in the assay. The ELISA signals obtained from non-affinity matured clones that showed a binding profile on yeast similar to that of the parental antibody M3_B11, were similarly low or even worse (data not shown).

**Figure 5:**
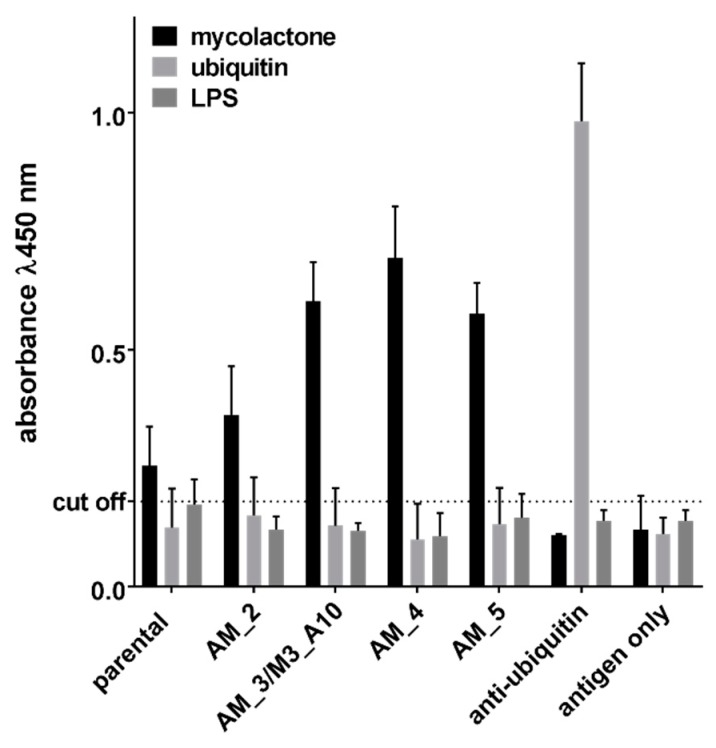
Detection activity of the scFv-Fc derived from individual affinity matured scFvs tested by ELISA against 300 nM of non-biotinylated mycolactone. Ubiquitin and LPS (300 nM) were used as negative control antigens and an anti-ubiquitin scFv-Fc derived from the same library was used as an assay positive control.

We performed an additional ELISA assay to ascertain whether the antibodies were able to bind to mycolactone that was not biotinylated. The results showed a very similar pattern to those obtained previously using biotinylated antigens (Figure 5). Antibodies also showed no binding to ubiquitin or LPS, used as negative controls, and directly coated to the ELISA plate.

### 2.3. Next Generation Sequencing (NGS) Analysis

Due to the relatively low number of identified binders obtained by traditional screening, we wanted to assess the entire diversity of the mycolactone-binding population displayed on yeast by NGS. We focused our analysis on the third heavy chain complementary determining region (HCDR3), which is generally considered to be the most important determinant of antibody binding, due to its position in the center of the antibody paratope, and its enormous diversity, compared to the other complementarity determining regions.

**Figure 6:**
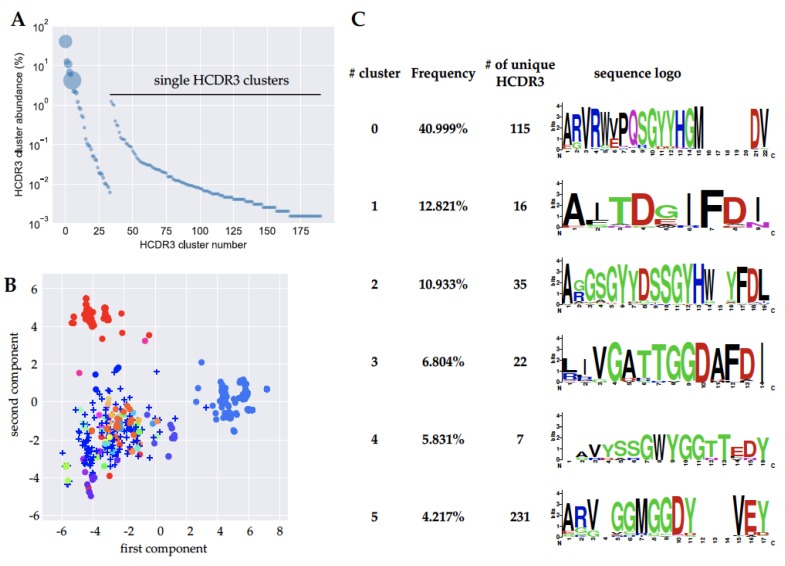
(**A**) Plot of anti-mycolactone HCDR3 antibody sequences plotted against the percentage abundance (*y*-axis). The bubble sizes are proportional to the number of different HCD3 sequences present in each cluster. (**B**) Plot of the first two components of the principal component analysis (PCA) performed using the Levenshtein distance of the HCDR3 amino acid sequences of sorted antibodies recognizing mycolactone. Different colors represent different clusters of HCDR3 identified. Clones represented as crosses did not cluster with any other sequence. (**C**) Clonotypic analysis of individual clusters showing the variability in each position of the HCDR3 sequence. The consensus sequences are generated from alignment of all HCDR3 sequences belonging to a given cluster. In some cases, there are a few HCDR3 sequences that contains additional amino-acids not present in most other sequences resulting in apparent ‘gaps’ in the consensus sequences.

We analyzed approximately 200,000 reads from MiSeq sequencing on the sorted output, which revealed 666 different HCDR3 sequences. For a better understanding of the effective functional diversity of these CDRs we calculated the Levenshtein distance between these HCDR3 amino acid sequences and applied a principal component analysis for visualization (Figure 6A). The plot revealed the presence of at least two regions of high density, suggesting clusters of HCDR3s. We then used a greedy iterative clustering method to group the HCDR3 amino acid sequences. We found 34 clusters containing more than one different HCDR3, plus 157 additional HCDR3s that did not cluster with any other sequences (Table S1). Interestingly, a large diversity was found in clusters 0 and 5, containing 115 and 231 different HCDR3 sequences, respectively (Figure 6B,C). Other clusters also showed modest diversity, such as cluster 1, 2, 3, 4, with 16, 35, 22, and 7 different sequences respectively. The functionality of these groups of clones remains untapped, since by random colony screening we have found and tested clones from just a few of them (0, 1, 2, 3, 4, 5, 13, and 14), leaving a large space for the exploration and development of new anti-mycolactone antibodies.

## 3. Discussion

In the present work, we describe an approach to generate recombinant human anti-mycolactone antibodies that is complementary to the one implemented by Dangy et al., [33], who showed that highly specific, monoclonal antibodies could be generated by immunization of mice using synthetic truncated mycolactone coupled to BSA as an immunogen. These murine antibodies demonstrated varying degrees of in vitro mycolactone neutralization, which in the best case prevented cellular apoptosis at an antibody:toxin molar ratio of 2.5:1. These results illustrate the potential of synthetic mycolactone derivatives to act as vaccines to prevent Buruli ulcer, and the possibility of developing new tools for research, diagnosis, prevention, and control of Buruli ulcer. The neutralizing antibodies obtained by Dangy et al. have the limitation of being derived from hybridomas, making them suboptimal for the treatment of human subjects, due to the possibility of anti-murine antibody responses [55]. Although it is feasible to obtain humanized derivatives of these murine antibodies to potentially treat Buruli ulcer, such strategy can be time consuming and does not always guarantee that the ‘converted’ antibodies will retain the same efficacy or might still trigger an immunogenic reaction [56,57]. For this reason, human antibodies, obtained from in vitro antibody libraries can provide a potentially better option for the treatment of Buruli ulcer, either by systemic or local administration.

In this publication, we demonstrate the preliminary generation of human recombinant antibodies recognizing mycolactone by direct selection on biotinylated complete mycolactone from a large naïve natural human antibody library. This result reinforces the value of in vitro display methods to select antibodies against toxic molecules that can be more difficult to use in traditional immunization. A first attempt (Selection 1) to isolate antibodies against mycolactone resulted in only a single clone; we speculated that this result was due to the very hydrophobic nature of mycolactone: even if some phage bind the molecule, its strong interaction with the plastic surface makes the rescue of bound phage during the selection step extremely difficult. When glass vials were implemented (Selection 2) we were able to rescue a total of 10 different antibodies, including the one already identified after the ‘plastic-based’ selections. Interestingly the only clone identified during the Selection 1 attempt, was the third most abundant clone (18%) of the final enriched population obtained by the Selection 2 approach, while the most abundant clone (30%) differed by only one amino acid (V instead of E) in the HCDR3. In general, the affinities of the selected antibodies were poor, especially when compared with those obtained against protein antigens from the same library (ranging from 2 to 100 nM), a finding that is consistent with results obtained against peptide and other small molecule targets [47].

For this reason, we tried to improve the affinity of the first identified clone binding mycolactone, found in Selection 1, by using error-prone PCR, and were able to obtain modest improvements. Interestingly, the sequences of the two best clones (~3-fold affinity improvement) were found to be identical to the best binder M3_A10 obtained after Selection 2, and closely related to M3_A10 differing by two additional amino acids in HCDR1.

The affinity maturation effort indicates the need to implement more sophisticated approaches, such as targeted CDR mutations [58] on the higher affinity antibodies already obtained. With further affinity maturation, it is expected that the affinities of these antibodies could be significantly improved, allowing their possible use in point of care diagnostic assays, perhaps in combination with those antibodies recently developed by traditional approaches [33].

Because of the traditional screening approach of picking random clones, sequencing and testing them might limit our capacity to identify all the potential binders selected during our campaigns against mycolactone. We implemented next generation sequencing (NGS) to allow a far deeper examination of the antibodies present in a selection output. The analysis was centered on the third heavy chain complementary determining region (HCDR3), generally considered to be the most important determinant of antibody binding, due to its position in the center of the antibody paratope [59], and its enormous diversity [60,61], compared to the other complementarity determining regions. Although a particular HCDR3 sequence does not necessarily predict specific binding [62], HCDR3 diversity is sufficiently great that it can be considered a ‘fingerprint’, and used to identify antibody clonotypes likely to bind to the target similarly. When we sequenced the entire selected antibody pool after Selection 2 by NGS, we found that a number of different clonotypes were present, and that our initial screening approach was only able to identify the most abundant ones, suggesting that a larger number of potential binders, possibly recognizing different regions of the mycolactone molecule, can be rescued from our selection attempts. We plan to rescue some of these different antibodies and test them against previously described synthetic mycolactone derivatives [33], to see whether they are able to compete with the murine monoclonal antibodies, or recognize alternative epitopes. Such a plethora of novel mycolactone antibodies represent the first important step towards the generation of rapid and robust diagnostic assays, where different antibodies recognizing different epitopes can guarantee better sensitivity and specificity.

## 4. Materials and Methods

### 4.1. Biotinylated Mycolactone

Biotinylated mycolactone was produced according to a modification of the protocol reported by Chany et al. [63]. To synthetic mycolactone A/B provided by Pr. Y. Kishi (1 mg, 1.35 μmol), a 0.01 M solution of NaIO_4_ (0.269 mL, 2.69 μmol) was added in a 1:1 mixture of THF/H_2_O (0.5 mL). The reaction was stirred and protected from light at room temperature for 1.5 h. Then, the reaction mixture was quenched by the addition of a 1 M aqueous Na_2_S_2_O_3_ solution and diluted with EtOAc. The organic layer was dried over anhydrous sodium sulfate and concentrated under reduced pressure. The crude residue was resuspended in MeOH (167 μL) before the addition of (+)-biotinamidohexanoic acid hydrazide (1.5 mg, 4.04 μmol) dissolved in DMSO (167 μL). The resulting mixture was stirred and protected from light at room temperature for 24 h. The reaction was then partitioned between water and EtOAc. The organic layer was washed with brine, dried over anhydrous sodium sulfate, and concentrated. The crude residue was purified by preparative TLC (elution with AcOEt/MeOH 8:2) to give biotinylated mycolactone (1.2 mg, 89%) and the corresponding sulfoxide (0.4 mg, 11%) as a yellowish oil and as a mixture of isomers that was not separated (*E*-Δ^4’-5’^/*Z*-Δ^4’-5’^ 1:1).

Determination of HPLC purity: Purity > 95% of biotinylated mycolactone was determined by HPLC (analytical C18 column (Eclipse XDB-C18), 3.5 μm, 3.1 × 30 mm, sample concentration: 0.2 mg/mL in CH_3_CN/H_2_O, injection: 5 μL, eluent: CH_3_CN/H_2_O 60:40 to 100:0, flow: 0.3 mL/min). LCMS t_R_-(biotinylated mycolactone) = 4.790 min, *m*/*z* 503.9 ([M + 2H]^2+^); tR-(biotinylated mycolactone-sulfoxide) = 2.704 min, *m*/*z* 511.9 ([M + 2H]^2+^).

### 4.2. Phage Display Selections

The first selection was carried out using the automated Kingfisher magnetic bead system [64] (Thermo Lab Systems), using a large naive phage display antibody library [44]. The library was obtained from 40 healthy donors by amplifying the V genes from the IgM B-cell pool, resulting in a collection of antibody fragments from a source of genes that is not biased toward specific antigens, but useful for selecting antibodies against all possible types of antigen structures. A measure of 5 μM of biotinylated mycolactone was used in each selection cycle, where the biotinylated mycolactone was incubated with the phage antibody library and the bound phages were captured using 2 × 10^7^ streptavidin-conjugated magnetic beads (Dynabeads M-280). After removal of the non-binding phage, the remaining phage particles were recovered from the beads by acid elution and used to infect F’ pilus-carrying bacteria (Ominmax-2T1, Thermo Fisher Scientific, Waltham, MA, USA). The phages were propagated and the selection cycle reiterated. Three rounds were performed in this selection. In a second phage selection, two rounds of phage selection were performed manually in screw top amber glass vials (Agilent Technologies) using the same technique described above with 500nM of biotinylated mycolactone in each selection round.

### 4.3. Yeast Display and Sorting of scFvs

After phage selection, the output of scFv clones were PCR amplified with specific primers introducing an overlapping region with the yeast display vector pDNL6 [46,50]. The vector and the amplification products were co-transformed into competent yeast cells to allow cloning by homologous recombination [65]. The yeast mini-libraries were further enriched for target-specific binders by applying two rounds of flow cytometry sorting (FACSAria, Becton Dickinson, San Jose, CA, USA), as described [65]. After induction, 2 × 10^6^ yeast cells were incubated with 1uM biotinylated mycolactone (first selection) and 500nM (second selection). Cells were labeled with streptavidin-Alexa-Fluor 633 to detect binding of biotinylated target antigens and anti-SV5-PE to assess scFv display levels. In the second sort, neutravidin-Alexa-Fluor 633 was used to replace conjugated streptavidin and eliminate the chance of sorting streptavidin binders. Yeast clones showing both antigen binding (Alexa-Fluor 633 positives) and display (PE positives) were sorted. The collected cells were grown at 30 °C for 2 days and induced for the next round of sorting at 20 °C for 16 h.

Final sorted outputs from both selections were transformed into bacteria (Ominmax-2T1) and plated for single colony isolation. Clones were picked from each selection and analyzed by Sanger sequence analysis, which provides the full-length sequence of the scFv clones and can determine the diversity of the enriched antibodies. In fact, the clones were determined to be unique based on the HCDR3 amino acid sequence. The first selection contained only one unique clone which was used as the template for affinity maturation. Unique clones identified from the second selection were expressed on yeast and affinity measurements were carried out by using the antibody displayed on the yeast and flow cytometry, according to published methods [66]. The output from the second selection was PCR amplified, gel purified, and prepared for next generation sequence analysis. All the described flow cytometry experiments were performed using the FACSAria (Becton Dickinson).

### 4.4. Affinity Maturation

Four rounds of affinity maturation using error prone PCR and yeast sorting were performed [67]. Decreasing biotinylated mycolatone concentrations were used between affinity maturation rounds (1 uM, 500 nM, 250 nM, and 125 nM). The parental clone, identified in the first selection, was used as the starting template for error prone PCR. The yeast expression vector and the error prone amplification product were co-transformed into competent yeast cells to allow cloning by homologous recombination, described above. Using flow cytometry individual yeast cells with positive mycolactone binding signal and scFv display were sorted, propagated, and subsequently used as template for additional affinity maturation rounds. The output from each affinity maturation round was analyzed by flow cytometry and the streptavidin-Alexa-Fluor 633 mean fluorescent value (mycolactone binding) were measured. The second and final affinity maturation rounds were transformed into bacteria (Ominmax-2T1), plated for single colony isolation, and analyzed by Sanger sequence analysis. Unique clones were determined by the full-length heavy chain amino acid sequence. Representative clones were expressed on yeast, affinity measurements calculated, and monoclonal antibodies produced.

### 4.5. Production of Monoclonal Antibodies (Yeast)

Unique clones were subcloned into the yeast expression vector pDNL9-HMR (Human Minibody for Recombination) to allow the expression and secretion of scFvs as human Fc fusions [68] into the culture supernatant. pDNL6 and pDNL9-HMR were designed to have compatible ends to promote in vivo homologous recombination of the PCR amplified yeast display library into the yeast expression vector. YVH10 yeast cells (provided by Prof. Dane Wittrup, Massachusetts Institute of Technology) were used for expression of monoclonal antibodies. Yeast antibody expression was performed following secretion protocols depicted in Wentz and Shusta [69] using SGT as induction media. Culture supernatants were used directly as reagents in western blots.

### 4.6. ELISA Screening of Antibodies

ELISA was performed by coating 96-well ELISA plates (Nunc) with 10 ug/mL neutravidin diluted in 1× phosphate-buffered saline (PBS), and incubating the plate overnight at 4 °C. Wells were blocked with 2% MPBS (1× PBS, 2% skim milk (*w*/*v*)) and subsequently incubated with 300 nM biotinylated mycolactone or ubiquitin as a negative control. After a rinse step, yeast undiluted supernatants were added to the wells. After one-hour incubation at room temperature, a further rinse step was performed, followed by HRP-conjugated anti-human Fc (Jackson ImmunoResearch, West Grove, PA, USA) diluted 1:5000 in PBS and incubated 1 h at room temperature. After a final rinse step, the immunocomplexes were revealed by adding TMB (Sigma-Aldrich, St. Louis, MO, USA) and reading the plate at 450 nm. ELISA with non-biotinylated mycolactone and ubiquitin and LPS as negative control was performed by directly coating 96-well ELISA plates (Nunc) with 300 nM of molecules overnight at 4 °C. Wells were blocked with 2% MPBS (1× PBS, 2% skim milk (*w*/*v*)) and, after a washing step, yeast undiluted supernatants were added to the wells and the ELISA completed following the procedure described above.

### 4.7. Next-Generation Sequencing

The VH regions of the antibodies obtained after Selection 2 were amplified with specific primers and submitted for Illumina MiSeq 2 × 250 bp sequencing (SeqMatic, Fremont, CA, USA). The CDR regions of these clones were identified using IgBlast. For HCDR3 analysis, in-house scripts were developed using Python3.7. Levenshtein distance was calculated using the package ‘distance’ and was normalized by the length of the smallest sequence. Sequences with less than 30% distance were clustered. Principal component analysis was performed using the function implemented at the package ‘scikit-learn’.

## Figures and Tables

**Table 1:** Complementarity determining regions (CDRs) of different mycolactone specific antibodies identified by phage and yeast display using the two selection strategies described in the text.

Clone ID	LCDR3	HCDR1	HCDR2	HCDR3	Abundance after Selection 1	Abundance after Selection 2
M3_B11	MQARQTPPT	GGTFSSYA	IIPIFGTA	ARVRWEPQSGYYHGMDVW	100%	18%
M3_A10	MQARQTPPT	GGTFSSYA	IIPIFGTA	ARVRWVPQSGYYHGMDVW	0%	30%
M3_E1	AAWDDSLNGPA	GYTFTSYG	YTFTSYG	ARVGGMGGDYVEYW	0%	20%
M3_B12	SSYSSSSSYV	GGTFSSYA	IIPIFGTA	LIVGATTGGDAFDIW	0%	16%
M3_B4	LLYYGGDWV	GGTFSSYA	IIPIFGTA	AAVGLDAFDIW	0%	4%
M3_C6	MQGTHWPPT	GGTFSSYA	IIPIFGTA	AITDGIFDIW	0%	4%
M3_A2	AAWDDRLNGVV	GGTFSSYA	IIPIFGTA	ARGSGYYDSSGYHWYFDLW	0%	2%
M3_C11	SSYAGSNGSV	GGTFSSYA	IIPIFGTA	AVYSSGWYGGTTEDYW	0%	2%
M3_E9	MQGTHWPPT	GGTFSSYA	IIPIFGTA	ARVAYYYGSGSYSFDYW	0%	2%
M3_B10	SSYSSSSSYV	GGTFSSYA	IIPIFGTA	AAADYYDSSGYYYGGVEEHW	0%	2%

**Table 2:** Sequences of the complementarity determining regions (CDRs) of different affinity matured mycolactone specific antibodies identified by yeast display after error prone PCR. CI represents the confidence interval.

Clone ID	HCDR1	HCDR2	HCDR3	Percentage Sequence Abundance			Yeast-Based Affinity	CI
				Selection Output	2 rds Affinity Maturation	4 rds Affinity Maturation		
parental	GGTFSSYA	IIPIFGTA	ARVRWEPQSGYYHGMDVW	91%	17%		470 nM	241–720
AM_1	GGTFSSYA	IIPIFGTA	ARVRWEPRSGYYHGMDVW		10%		360 nM	260–450
AM_2	GGTFSSYA	IIPIFGTA	ARVRWVPRSGYYHGMDVW		7%		345 nM	181–676
AM_3/M3_A10	GGTFSSYA	IIPIFGTA	ARVRWVPQSGYYHGMDVW		10%	20%	149 nM	71–271
AM_4	GGAFSRYA	IIPIFGTA	ARVRWVPQSGYYHGMDVW		29%	54%	145 nM	69–298
AM_5	GGTFSRYA	IVPIFGTA	ARVRWVPQSGYYHGMDVW		24%	16%	212 nM	159–324

## Supplementary Materials

The following are available online at https://www.mdpi.com/2072-6651/11/6/346/s1, Table S1: List of HCDR3 clusters found after selection against mycolatone.
